# 境外授权许可引进抗肿瘤新药临床研发策略的审评考虑

**DOI:** 10.3779/j.issn.1009-3419.2022.101.27

**Published:** 2022-07-20

**Authors:** 肖 赵, 瑞敏 郝, 昕 仝, 丽敏 邹, 凌 唐, 虹 张, 琳 夏, 志敏 杨

**Affiliations:** 100022 北京，国家药品监督管理局药品审评中心 Center for Drug Evaluation, National Medical Products Administration, Beijing 100022, China

**Keywords:** 授权许可, 抗肿瘤新药, 研发策略, 桥接研究, License-in, Anti-tumor drug, Research and development strategy, Bridging study

## Abstract

随着我国创新药产业的蓬勃发展，授权许可引进逐渐成为创新药企的重要研发模式。不同研发阶段引进的药物，在中国的研发策略有所不同。企业需全面整理药物已产生的境外临床数据，对临床药理学、安全性、有效性和种族敏感性进行详细的分析。应基于上述数据和分析的结果制定合理的临床研发策略。我们鼓励引进中国境内真正未满足临床需求的优质药物，尽可能在研发的早期阶段引进，以实现国内外的同步研发。新药引进后的临床研发策略对于药物能否顺利上市尤为重要，企业应遵循国家药品监督管理局已发布的指导原则和相应的临床指南进行临床试验设计，积极与监管部门进行沟通。

## Abstract (en)

With the boom of China's innovative pharmaceutical industry, licensing-in model has gradually become an important research and development model for innovative pharmaceutical companies. The in-licensed drugs at different stages need different research and development (R & D) strategy in China. The pharmaceutical companies take the responsibility to comprehensively collate the oversea clinical data and conduct a detailed analysis of clinical pharmacology, safety, efficacy and ethnic sensitivity. Clinical R & D strategy should be made based on the results of the above data and analysis. We encourage high-quality drugs which fill unmet clinical needs licensed in, and as early as possible, so as to conduct multi-regional clinical trials (MRCTs). The clinical R & D strategy in China is particularly important for the drug's approval. Guidelines published by the National Medical Products Administration (NMPA) and clinical associations should be followed. Communications about clinical R & D strategy with Center of Drug Evaluation (CDE) are encouraged.

## 1 背景

近年来，随着药物研发全球化进展，越来越多的国内制药企业选择采用新药授权许可引进（license-in）模式丰富自身产品线。据不完全统计，仅2021年，中国制药企业的自境外引进的项目就超过100起，其中近一半项目为抗肿瘤药物。

境外引进的药物可分布于不同的研发阶段，其中一半左右的药物处于临床前阶段，10%左右的药物已经开展境外关键注册研究，10%左右的药物已完成境外监管机构要求的关键注册研究或已经获得境外监管机构的上市批准。处于不同研发阶段的药物被国内企业引进后在中国的研发策略也有所不同。

本文以license-in的抗肿瘤新药为例，将我们在审评中遇到的问题及思考撰写成文，以期为企业研发策略提供参考，以促更高效的研发，更早地将更优质的药物引进中国，以惠及中国患者。

## 2 总体考虑

License-in模式的主要优势在于可借鉴国外已有的研究成果，提高研发效率；可以做到与国外几乎同步的研发进度，从而使产品更早地在国内上市。在研发的不同阶段引进新药对引进企业来说各有优劣。处于研发早期的药物竞争压力小，但研发周期长、不确定因素多、失败的风险高，对引进企业临床研发能力要求较高。已完成境外关键注册研究的药物，临床研发已较为成熟，不成药的风险大大降低，但竞争较大，且在监管方面有可能存在一些问题，如中国患者信息的缺失、已完成关键研究设计不符合中国临床实践或监管要求、需进行重复研究、浪费临床研究资源等。

无论在研发的任何阶段对药物进行引进，前期数据均在境外产生，应遵循国家药品监督管理局和人用药品技术要求国际协调理事会（The International Council for Harmonization of Technical Requirement for Pharmaceuticals for Human Use, ICH）制定的有关境外数据的相关指导原则，如《ICH E5（R1）：接受国外临床试验数据的种族因素》^[1]^、《ICH E17：多区域临床试验计划与设计的一般原则》^[1]^、《接受药品境外临床试验数据的技术指导原则》（2018年）^[2]^、《境外已上市境内未上市药品临床技术要求》（2020年）^[3]^等。除遵循上述指导原则外，临床研发还需遵守一般药物的研发要求。

## 3 中国境内开展的临床研究设计

不同研发阶段引进的药物，在中国的研发策略有所不同。企业需全面整理药物已产生的境外临床数据，对临床药理学、安全性、有效性和种族敏感性进行详细的分析。企业应基于上述数据和分析的结果制定合理的临床研发策略。对于license-in模式的药物要“借鉴”境外数据，而非“依赖”境外数据，开发模式也不仅限于全球同步，还可以根据中国临床实践的需求和特点在中国设计独立的研发策略。对于license-in产品在中国的研发策略，鼓励引进企业积极与药审中心进行探讨。

境外关键注册研究开始和完成是影响中国境内临床研发策略的关键时间点。根据境外开展首次人体研究、境外关键注册研究开始及完成可将license-in药物分为4类：未开展人体研究、境外关键注册研究尚未开始、境外关键注册研究已开始但尚未完成、境外关键注册研究已经完成（包括已有境外监管机构批准上市）。对于在不同研发阶段引进的药物，在中国境内的研发策略有不同的考虑。

### 3.1 在未开展临床试验阶段引进

此种情况可在国内外进行同步研发，也可考虑在中国进行独立开发。

### 3.2 探索研究阶段引进

对于尚处于探索阶段的药物，如正在探索2期推荐剂量（recommended phase 2 dose, RP2D）或在不同瘤种中探索初步有效性和安全性，引进后尽可能与境外进行同步研发，减少重复研究。

#### 3.2.1 境外尚在剂量爬坡阶段可同步在境内外开展剂量爬坡研究

如境外已获得初步安全性和临床药理学数据，可作为中国剂量爬坡研究的参考来制定国内爬坡研究的起始剂量。如有足够的境外人体数据可参考，中国研究的起始剂量不必依据临床前动物实验数据设计非常低的起始剂量。

如下2种情况：①境外有东亚患者数据，经种族敏感性分析，不存在种族差，且药物安全性风险较小，在境内开展的临床研究，中国患者的剂量爬坡可采用境外正在探索的剂量作为起始剂量，同步爬坡；②如经种族敏感性分析，种族间存在差异，或境外无东亚患者数据，通常采用低于境外正在探索的剂量作为中国患者的起始剂量。

由于剂量爬坡阶段每个剂量组入组患者例数较少，且研究过程中可能存在未知的安全性风险，需要研究者和企业迅速决策，为便于分析和降低风险，建议在国内外开展单独的剂量爬坡队列。

#### 3.2.2 境外研究已经获得RP2D

如境外的剂量探索研究已结束，已有充足的数据证明在境外人群中可确定RP2D。RP2D的确定通常有几种方式，细胞毒药物一般根据最大耐受剂量（maximum tolerated dose, MTD）确定RP2D，分子靶向或免疫治疗药物一般根据最佳生物效应剂量确定RP2D。在中国进行临床研究可充分参考上述数据，结合种族敏感性、药物代谢特点和作用机制等因素综合判断境外人群的RP2D是否适用于中国人群。

有如下3种情况：①通过MTD确定RP2D的药物，通常需在中国进行简化的剂量探索研究；根据境外获得的耐受性数据，选取2个-3个剂量组进行剂量爬坡研究；②通过最佳生物效应剂量确定RP2D的药物，如药物安全窗宽，且种族不敏感，可不用在中国患者中再开展剂量探索研究；③通过最佳生物效应剂量确定RP2D的药物，如有安全性担忧或种族敏感性不确定需在Ⅱ期/Ⅲ期研究前增加安全性导入研究。

#### 3.2.3 关键研究的设计

中国患者的RP2D确定后可参考ICH E17指导原则进行多区域临床研究（multi-regional clinical trial, MRCT）。

后续关键研究可能出现不同的情形：①中国患者RP2D与国外患者不同，如可充分解释在中国和境外不同地区的不同剂量可产生同样的有效性和安全性，可开展MRCT，MRCT的结果可合并处理；如采取不同剂量预期可能对有效性或安全性产生影响，实施MRCT可能会带来一些问题，可考虑在中国实施单独的关键研究；②国际间研发相同的适应证，需考虑各地区临床实践及监管要求是否相同，如研究人群、对照选择、主要终点等研究设计的关键点需满足各监管机构的要求；③如拟在境内外进行不同适应症的研发，需提供充分的理由。

### 3.3 关键研究已开始但尚未完成时引进

境外关键研究已开始入组患者，此时引进中国，是否可在中国直接开展关键注册研究，需从以下几个方面进行评估。

#### 3.3.1 评估RP2D是否适合中国患者

对于中国患者RP2D的评估，需要申请人提供充足的数据，具体要求可参考ICH E5和《接受药品境外临床试验数据的技术指导原则》（2018年）。如数据充分，证明中国患者与国外患者RP2D相同，且中国患者不存在安全性担忧，则可在中国直接开展关键MRCT，需在MRCT期间获得中国患者PK数据。如数据不充分或当前数据提示中国患者不适用国外研究的RP2D，需参考3.2.2部分的要求，完成中国患者的剂量探索研究。

#### 3.3.2 评估是否可同步开展关键研究

确定中国患者的给药剂量后，需对是否可同期开展MRCT进行评估。如中国地区开展关键研究的时间已明显落后于境外，无法开展MRCT，则可在中国境内开展桥接研究。

#### 3.3.3 评估关键研究设计是否符合中国监管要求及临床实践

境外已经开展的MRCT满足境外监管机构的要求和临床实践，也应符合中国的监管要求和医疗实践。虽然近些年抗肿瘤药物的研发多为国内外同步研发，国内外临床实践趋于统一，但某些领域仍然存在临床实践的差异。对照药应能反映中国可获得的最佳治疗，主要终点和中国患者的样本量应满足我国的监管要求。

### 3.4 关键研究已完成后引进

境外关键研究已完成的产品的后续开发策略，需综合风险获益、临床需求和种族敏感性分析等因素进行全面评估。对于境外数据的接受程度可参考《接受药品境外临床试验数据的技术指导原则》（2018年）、《境外已上市境内未上市药品临床技术要求》（2020年）两个指导原则。

对于境外关键研究显示生存获益显著的药物，境外关键研究设计符合我国临床实践和监管要求，经分析不存在种族差异，可在中国采取桥接研究的策略。

如生存获益不显著、不明确或提示可能存在安全性问题，或境外关键研究设计不符合我国临床实践或监管要求，需按新药要求在中国开展新的确证性研究。

如果境外研究已发现明确的严重安全性风险，不建议在中国境内继续研发。

如属于临床急需药物，用于治疗极罕见的恶性肿瘤，预期很长时间内难以完成国内的注册研究，由于存在巨大的未满足的临床需求，经评估获益远远超出当前的标准治疗且不存在种族差异，可完全接受境外数据，免除上市前临床研究。

根据境外研究数据的情况，可能还需补充其他类型的研究，如：剂量探索研究、药代动力学-药效学研究、安全性研究等。 

## 4 审评考虑

为了满足患者的临床需求，企业在引进项目时应以临床价值为导向。鼓励在药物研发的早期进行引进，实现国内外同步研发。对于在境外停止研发的产品，后续仅在中国进行研发的情况，企业需提供充分的数据和解释。对于同类产品，如在境外的临床研究中显示出了疗效差异，在中国的研发采取统一的监管标准。

### 4.1 对临床价值的考量

我们鼓励企业引进优质、先进的药品，以满足中国患者的临床需求。对产品的评价立足于解决当下中国临床实践中未满足的需求，并非所有在境外上市的药品均需引入中国。企业应根据产品机制、拟解决的临床问题，选择性引进。对于临床价值的评价一般会综合以下几个方面考虑：①机制创新性，如first in class；②满足临床急需或填补领域空白；③临床优势明显，如best in class。基于上述考虑，不建议引进国内已有很多在研同类药物的同质化产品；有些产品历史久远，临床实践早已发生变化，此类产品不建议再进行引进。

### 4.2 符合中国的监管要求

不论license-in产品在境外处于何种研发阶段，拟在中国获批，关键研究设计必须符合中国的监管要求。国内外监管机构的注册要求可能存在差异，因此积极地与药审中心进行沟通是非常必要的。如用于经一线治疗失败的晚期或转移性胆管癌患者的药物，美国食品药品监督管理局接受安慰剂作为对照、无进展生存期为主要终点的关键研究设计。而此类患者在临床实践中有多种化疗组合或单药可作为治疗选择，生存期短于1年。拟在中国进行注册的产品针对此类患者的关键临床研究设计应采取研究者选择的化疗作为对照，总生存期作为主要研究终点。

另一个值得关注的问题是境外license-in产品引进后存在一些特殊情况。如某些产品在引进后，境外停止研发，后续仅在中国研发上市。这导致我国对早期境外数据无法进行核查，境外停止研发后，也无其他境外机构对其数据进行监管。此种情形下，license-in产品早期数据的真实性无从考证，仅可作为参考；如果临床数据证据链不清晰，需要提高监管标准，在中国境内补充开展前期研究。又如license-in产品在境内外进行不同适应证研发，在中国进行申报时不能仅提供拟在中国研发适应证的临床数据，需提供境外完整的研究数据。

近年来境外关键研究完成后或境外监管机构已经批准后引进的产品越来越多，引进的企业通常期望产品可免除上市前临床研究。由于缺乏中国患者的临床数据，引进的产品质量参差不齐，有些产品甚至因安全性问题在国外已经停止研发或撤市，这些产品在中国研发对于监管机构存在巨大挑战。企业应全面掌握产品在境外的研发情况，对产品既往与境外监管机构的沟通交流情况进行详细梳理，并将相关材料在与药审中心的沟通中提交，企业需确保资料的真实性和全面性。

### 4.3 桥接研究的考虑

在中国实施ICH E17前，很多进口产品通过桥接研究的方式在中国获批上市。随着我国ICH系列指导原则的全面实施，中国有充分的条件与国外同步开展MRCT。我们建议license-in产品尽早引进，在剂量探索阶段即进行国内外同步研发。但部分license-in产品在引进时已处于临床研发阶段的后期，无法进行MRCT，某些符合条件的产品可通过桥接研究在中国上市注册。

桥接研究需在种族敏感性指导下开展，通过桥接数据将国外临床数据外推到中国。桥接研究通常采用较小规模的与国外关键研究设计相似的试验。以临床终点为研究指标的桥接研究通常采取一致性趋势来判断不同地区间的相似性。这在实际审评过程中带来了一些问题。如同类（同靶点）有多个产品在进行研发，不同产品间疗效数据可能存在差异。如针对某适应证的首个license-in产品A在境外上市采取了单臂关键研究，主要终点客观缓解率（objective response rate, ORR）为55%（95%CI: 47%-63%）。对于A产品的桥接研究，主要终点的要求为在中国的关键研究ORR点估计不低于47%。如B产品在境外采取了同样的上市策略，ORR为45%（95%CI: 38%-54%），疗效不及A产品。如此时B产品采取与A产品同样的桥接研究设计策略，则导致了疗效越差的产品在中国的注册要求越低的尴尬情况。因此，对于有多个同类药物在研发的情况，境外研究数据已显示疗效存在差异，在中国进行桥接研究时采用统一标准的监管要求。

## 5 总结

随着我国创新药产业的蓬勃发展，license in逐渐成为创新药企的重要研发模式。我们鼓励引进中国境内真正未满足临床需求的优质项目，谨慎对待赛道已过度拥挤的项目。新药引进后的临床研发策略对于药物能否顺利上市尤为重要，企业应遵循国家药品监督管理局已发布的指导原则和相应的临床指南进行临床试验设计。License-in药物尽可能在研发的早期引进，以实现国内外的同步研发。
